# Transmission, Tropism, and Biological Impacts of Torix *Rickettsia* in the Common Bed Bug *Cimex lectularius* (Hemiptera: Cimicidae)

**DOI:** 10.3389/fmicb.2020.608763

**Published:** 2020-12-23

**Authors:** Panupong Thongprem, Sophie E. F. Evison, Gregory D. D. Hurst, Oliver Otti

**Affiliations:** ^1^Institute of Infection, Veterinary and Ecological Sciences, University of Liverpool, Liverpool, United Kingdom; ^2^Faculty of Medicine & Health Sciences, University Park, Nottingham, United Kingdom; ^3^Animal Population Ecology, Animal Ecology I, Bayreuth Center for Ecology and Environmental Research (BayCEER), University of Bayreuth, Bayreuth, Germany

**Keywords:** torix *Rickettsia*, *Cimex lectularius*, endosymbiont, maternal inheritance, symbiosis

## Abstract

The torix group of *Rickettsia* have been recorded from a wide assemblage of invertebrates, but details of transmission and biological impacts on the host have rarely been established. The common bed bug *(Cimex lectularius)* is a hemipteran insect which lives as an obligatory hematophagous pest of humans and is host to a primary *Wolbachia* symbiont and two facultative symbionts, a BEV-like symbiont, and a torix group *Rickettsia*. In this study, we first note the presence of a single *Rickettsia* strain in multiple laboratory bed bug isolates derived from Europe and Africa. Importantly, we discovered that the *Rickettsia* has segregated in two laboratory strains, providing infected and uninfected isogenic lines for study. Crosses with these lines established transmission was purely maternal. Fluorescence *in-situ* hybridization analysis indicates *Rickettsia* infection in oocytes, bacteriomes, and other somatic tissues. We found no evidence that *Rickettsia* infection was associated with sex ratio distortion activity, but *Rickettsia* infected individuals developed from first instar to adult more slowly. The impact of *Rickettsia* on fecundity and fertility resulted in infected females producing fewer fertile eggs. However, we could not find any evidence for cytoplasmic incompatibility associated with *Rickettsia* presence. These data imply the existence of an unknown benefit to *C. lectularius* carrying *Rickettsia* that awaits further research.

## Introduction

Symbioses between insects and bacteria are both common and important. Bacterial symbionts impact on the biology of their host individual, and also by extension affect the ecology and evolution of their host (Mitter et al., 1988; Sudakaran et al., 2017). Effects are diverse, with beneficial effects ranging from nutritional (e.g., anabolism or digestion), protection against multiple different forms of natural enemies and also against xenobiotics (Hosokawa et al., 2010; Werren, 2012; Hendry et al., 2014). Parasitic interactions are also known, with the maternal inheritance of the symbiont selecting for sex ratio distortion activity (Hurst and Frost, 2015).

The genus *Rickettsia* (alpha-proteobacteria) has emerged as an important associate of insects. Members of this genus were classically considered as causative agent of arthropod-borne rickettsioses, which threaten livestock and human health (Gaon and Murray, 1966; Gross, 1996). These zoonotic pathogens are endosymbionts of ticks, mites, fleas and lice (Azad and Beard, 1998). Following a bite, the bacteria disseminate into the blood of mammals where it causes disease such as typhus and spotted fever (Gaon and Murray, 1966; Azad and Beard, 1998). In 1994, however, studies of ladybirds (*Adalia bipunctata*) let to the discovery that *Rickettsia* can exist strictly as vertically transmitted endosymbionts of arthropods (Werren et al., 1994), with no mammalian transmission. These *Rickettsia* symbionts are known to present a significant selection pressure on their insect hosts by, for example, altering reproductive success and distorting sex ratio by inducing male-killing in ladybirds (Werren et al., 1994) and parthenogenesis in a parasitoid wasp (Giorgini et al., 2010). Unlike *Cardinium, Rickettsiella* and *Wolbachia, Rickettsia* has never been implicated in causing cytoplasmic incompatibility in arthropods (Zabalou et al., 2004; Gotoh et al., 2007; Rosenwald et al., 2020). With regard to host fitness, some *Rickettsia* strains may aid host defense against pathogens (Hendry et al., 2014).

In 2002, a new group of *Rickettsia* were discovered during research on *Torix tagoi* leeches. This *Rickettsia* infection was associated with changes to leech growth and development, with infected individuals larger than uninfected comparators (Kikuchi et al., 2002; Kikuchi and Fukatsu, 2005). The *Rickettsia* was a sister group to all other *Rickettsia* described previously, and the clade were named “torix *Rickettsia”* (Kikuchi et al., 2002). Torix *Rickettsia* have since been found across multiple arthropod taxa and seem to be widespread and especially common in species associated with freshwater [e.g., *Culicoides* midges (Pilgrim et al., 2017), dytiscid water beetles (Küchler et al., 2009), Odonata (Thongprem et al., 2020a), and Amphipoda (Park and Poulin, 2020)], but have also been detected in some terrestrial arthropods, e.g., Araneidae (Goodacre et al., 2006), Siphonaptera (Song et al., 2018), and Hemiptera (Wang et al., 2020). Whilst we now understand symbioses between invertebrates and torix *Rickettsia* are common, much less is known of their biological significance. This knowledge deficit arises largely from the lack of a good laboratory model systems in which inheritance and biological impact can be measured.

In this study, we characterize the patterns of inheritance and biological impact of torix *Rickettsia* in the common bed bug (*Cimex lectularius*). This species is in the order Hemiptera, belonging to the family Cimicidae, all members of which are ectoparasites of warm-blooded animals (Usinger, 1966). *Cimex lectularius* is a human parasite and its global pest status has medical, social and economic impacts (Hwang et al., 2005; Ribeiro and Valenzuela, 2011). Being an obligate haematophage, *C. lectularius* have evolved a special organ called a “bacteriome” that harbors *Wolbachia*, which live as a primary endosymbiont and synthesize B-vitamins to supplement the host's blood diet (Hosokawa et al., 2010). In some individuals, *Wolbachia* is found alongside a facultative gamma-proteobacterium, also known as BEV-like symbiont (Hosokawa et al., 2010; Meriweather et al., 2013). The impact of this symbiont is not currently understood.

A recent PCR-based screen by Potts et al. (2020) revealed *Rickettsia* associated with natural populations of *C. lectularius* in both the UK and the USA. Partial citrate synthase gene (*gltA*) sequences showed that this strain is closely related to the *Rickettsia* found in the flea *Nosopsyllus laeviceps* (Song et al., 2018). Recent work by ourselves has indicated *C. lectularius* genomic DNA samples from Duron et al. in 2008 (Duron et al., 2008), also carried a *Rickettsia* symbiont. The analysis revealed the presence of torix *Rickettsia* in multiple individuals from one laboratory strain, F4.

To explore the torix *Rickettsia* association with *C. lectularius*, we undertook PCR assays to investigate the genetic diversity and prevalence of these *Rickettsia* strains in *C. lectularius* cultures originally collected from various locations across the UK, Europe and Africa. We isolated two laboratory lines where *Rickettsia* infection had segregated, and used these to analyse the transmission, tissue tropism and biological impacts of *Rickettsia* infection. These results indicate a maternally inherited symbiont with a broad somatic/germline distribution, that does not impact host sex ratio or generate cytoplasmic incompatibility. Impacts on host development and reproduction are minor, indicating there likely to be an as yet unelucidated impact on the host.

## Materials and Methods

### Prevalence of Torix *Rickettsia* in *C. lectularius* Populations and Cimicid Allies

One male and one female adult bed bug from each of 21 lab populations maintained at the University of Bayreuth (Table 1) were sent in 2.0 ml absolute ethanol tubes (one pair/tube) to the lab at the University of Liverpool for DNA extraction. These populations were collected from different areas of Europe and Africa in different years (Table 1). Two populations are of unknown origin in the wild. One of these has been maintained at the Universities of Bayreuth and Sheffield for >20 years and before that for >40 years at the London School of Hygiene and Tropical Medicine. The other population was received from Bayer (Germany) in 2006.

**Table 1:** *Rickettsia* infection in *Cimex lectularius* stock populations in Bayreuth lab.

**Population name**	**Origin of place**	**Year established in Sheffield**	**Male/Female (M/F) positive**
H1	Budapest, Hungary	2010	neither
C1	Coventry, UK	2010	neither
Bayer	Bayer Environmental Science, Germany	2006	neither
S1*	London School of Tropical Medicine	1996	(M)/F
London heavy	London, UK	2006	neither
F11	London, UK	2006	neither
F4*	London, UK	2006	M/F
F10	London, UK	2006	neither
YMCA	London, UK	2010	neither
K12	Nairobi, Kenya	2008	M/F
K4	Nairobi, Kenya	2008	M/F
K22	Nairobi, Kenya	2010	M/F
K25	Nairobi, Kenya	2010	M/F
K26	Nairobi, Kenya	2010	M/F
K19	Nairobi, Kenya	2010	M/F
K20	Nairobi, Kenya	2010	M/F
K7	Nairobi, Kenya	2008	M/F
K5	Nairobi, Kenya	2008	M/F
K30	Nairobi, Kenya	2010	M/F
BG1	Sofia, Bulgaria	2010	(M)/F
K17	Watamu, Kenya	2010	neither

*Males in the brackets (M) indicate the infection status were detected later when these populations were screened with more individuals. Asterisks after a population name indicate that these populations were selected for transmission mode experiment and established as the isofemale lines. Except for Bayer and S1, all populations originated from independent infestations in private homes or youth hostels (Populations H1 and YMCA)*.

The samples were rinsed with absolute ethanol and left at room temperature until the samples dried. To avoid contamination with gut microbes, bed bugs were decapitated with sterilized forceps and only the head and/or the upper part (from the head to the thorax, including legs) taken for DNA extraction. Genomic DNA was extracted from the selected body part using Promega Wizard® Genomic DNA Purification kit (A1120, Promega, UK) and DNA dissolved with 100 μl of molecular water and stored in −20°C for the future use.

In addition, DNA template was obtained from various species of cimicids from the recently published bed bug phylogeny by Roth et al. (2019) (Table 2). For each species, we received 10 μl extracted genomic DNA in 0.2 μl tubes from Steffen Roth (University Museum of Bergen, Norway) and Klaus Reinhardt (TU Dresden, Germany), which we stored in −20°C until used. Voucher specimens of some species are stored in the collection of the University Museum of Bergen (ZMNB), Norway.

**Table 2:** *Rickettsia* infection in cimicid allies.

**Subfamily**	**Species**	***N***	**Locality**	**Year of collection**	**Infection**
**Afrocimicinae**	***Afrocimex constrictus***	**3**	**Kenya**	**2005**	**+**
Primicinae	*Bucimex chilensis*	1	Chile	2013	–
	*Primicimex cavernis*	1	Mexico	2015	–
Haematosiphoninae	*Acanthocrios furnarii*	1	Brazil	2010	–
	*Cimexopis nyctalis*	1	USA	2016	–
	*Cyanolicimex patagonicus*	1	Argentina	2003	–
	*Hesperocimex coloradensis*	1	Los Alamos County, N.Mex.	1971	–
	*H. sonorensis*	1	Mexico	2017	–
	*Psitticimex uritui*	1	Argentina	2008	–
	*Ornithocoris pallidus*	1	USA, South Carolina	2010	–
Cimicinae	*Cimex hemipterus*	1	Taiwan	Before 1966	–
	*C. hirundinis*	1	Switzerland	N/A	–
	*C. pipistrelli*	1	Hanau, Germany	2004	–
	*Paracimex borneensis*	1	Borneo	2015	–
	*P. inflatus*	1	Kavieng, Papua New Guinea	1996	–
Cacodminae	*Aphrania elongata*	1	Senegal	2012	–
	*A. vishnou*	1	Phnom Penh, Cambodia	1952	–
	*Cacodmus villosus*	1	Kenya	2005	–
	*Leptocimex boueti*	1	N/A	N/A	–
	*L. duplicatus*	1	Israel	2002	–
	*Loxapsis malayensis*	1	Tasik Bera, Pahang, Malaysia	1962	–

*The infection found in all individuals of Afrocimex constrictus but absent in other tested species*.

Initially, all DNA samples were checked for their quality using the invertebrate mtDNA barcoding primers C1J_1718 (Simon et al., 1994) and HCO_2198 (Folmer et al., 1994) (see Supplementary Table 1) that amplified a fragment of approximately 380 bp of the cytochrome oxidase subunit I (*COI)* gene of *C. lectularius*. For the samples that passed quality control, we assessed the presence of *Rickettsia* infections using two *Rickettsia*-specific primer pairs, targeting 16S rRNA gene, and *gltA*, the citrate synthase gene (16SrRNA: Ri170_F and Ri1500_R Küchler et al., 2009; *gltA*, RiGltA405_F and RiGltA1193_R Pilgrim et al., 2017, Primer sequences, cycling conditions and expected amplicon size given in Supplementary Table 1). These primer pairs for *Rickettsia* are specific for the currently known *Rickettsia* groups and do not cross amplify other Rickettsiales (Küchler et al., 2009; Pilgrim et al., 2017; Thongprem et al., 2020b). Amplicons were retained for sequencing (see below).

Where we observed only one of the two individuals within a line to be infected with *Rickettsia* in this initial screen, we screened more individuals (4–10 samples, mixed males and females) to verify the infection status across a wider range of individuals. This screen additionally allowed us to determine if there were any sex-specific patterns of symbiont infection typical of a sex ratio distorting symbiont.

### Relatedness of Strains

The *16S rRNA* and *gltA* amplicons from PCR assays were cleaned with the ExoSAP-IT kit (E1050, New England Biolabs, US) and Sanger sequenced. The sequence chromatograms were trimmed and edited in UGENE (Okonechnikov et al., 2012). All the sequences were exported to fasta format and searched against other *Rickettsia* strains on NCBI database to find close relatives ascertained by BLAST homology. The sequence of these markers from closely related *Rickettsia* strains from other invertebrate hosts were retrieved, and the relatedness within the torix group estimated. Other *Rickettsia* strains from other clades, e.g., *Rickettsia bellii* and vertebrate pathogens were selected to represent the sister group to the torix clade. *Occidentia massiliensis* was use as the outgroup for both topologies. All the selected sequences were aligned with *Rickettsia* sequences in this study using MUSCLE algorithm with its default setting in MEGA X (Kumar et al., 2018; Stecher et al., 2020). The ML phylogeny for both genes were estimated in MEGA X with 1,000 rapid bootstrap replicates under T92+I and K2+I model for *gltA* and 16S gene, respectively.

### Transmission Mode Experiment

To investigate the vertical transmission mode of torix *Rickettsia*, we used two bed bug lab populations, S1 and F4, both of which we had found to contain a mix of infected and uninfected individuals. We randomly selected males and females to establish 55 and 49 mating pairs for S1 and F4, respectively, from which we reared offspring. The parents and 5–10 randomly selected first instar nymphs per cross were screened for torix infection status using the PCR assays as described above. First-instar nymphs were tested individually to gain insight into vertical transmission efficiency, and whole bodies were used for template. We then assessed the impact of parental infection status (mother infected, father infected) on progeny infection status.

### Isofemale Lines and Bed Bug Culture

Based on the infection status of offspring from the transmission mode experiment, we established four *Rickettsia*-free (R–) and four *Rickettsia*-infected (R+) isolines for each of the F4 and S1 populations. These isofemale lines of known *Rickettsia* infection status were then kept under constant conditions, in a CT room at 26 ± 1°C, at about 70% relative humidity with a cycle of 12L:12D. We additionally tested all isofemale lines for the BEV-like bacterium infection using a PCR assay as described in Degnan et al. (2011) (Supplementary Table 1). New generations were set up regularly, i.e., at a 6–8-week interval. Each new generation was started with randomly picked virgin female and virgin male. All bed bugs were maintained in the CT room with the conditions described as above. All individuals in our study were virgin prior to experiments. The feeding, maintenance and generation-of-virgin-individuals protocols follow Reinhardt et al. (2003).

### Fluorescence *in situ* Hybridization (FISH)

To localize the torix *Rickettsia* and other symbionts within the *C. lectularius* body, we used the FISH technique adapted from Sakurai et al. (2005). We investigated the bacteriome and reproductive tissue in virgin male and female adults, as well as the whole body of first instar nymphs from the R+ and R– F4 and S1 lines. Tissues were dissected in 0.5M PBS at pH 7.4 and preserved immediately in Carnoy's solution (chloroform: ethanol: glacial acetic acid = 6:3:1) overnight. Bedbug nymphs were preserved in the solution without dissection. All tissue samples were cleared by incubating in 6% H_2_O_2_ in ethanol for 12 h, save for the whole-body nymph that was incubated at least 24 h or until the body was transparent. We then used a tungsten micro-needle to make micropores in the nymph cuticle to allow the fluorescence probes to pass through the cuticle during the hybridization step. The samples were hybridized by incubating the tissues overnight in a hybridization buffer (20 mM Tris-HCl pH 8.0, 0.9 M NaCl, 0.01% Sodium dodecyl sulfate 30% formamide) with 10 pmol/ml of rRNA specific probes for *Rickettsia* (Perotti et al., 2006), *Wolbachia* (Hosokawa et al., 2010), and Gamma proteobacteria (BEV-like symbiont) (Hosokawa et al., 2010) (probe sequences in Supplementary Table 1). We also used nuclei fluorescence staining, Hoechst 33342 (H1399, Invitrogen, Carlsbad, USA), to visualize bed bug tissues. After incubation, tissues were washed in buffer (0.3 M NaCl, 0.03 M sodium citrate, 0.01% sodium dodecyl sulfate) and mounted onto a slide using VECTASHIELD® Antifade (H-1000, Vectorlabs, UK) as a mounting medium. Slides were then observed under a confocal microscope, 880 Bio AFM (on 880 LSM platform, ZEISS, Germany).

### Development Time and Sex Ratio Produced by *Rickettsia* Infected and Uninfected Individuals in a Common Garden Experiment

A 7-day-old virgin male and female from each isofemale line were placed together in a pot and allowed to mate. Individuals from both sexes were fully fed as post-eclosion adults, and also immediately prior to mating, which ensured gamete production until day 7. Once offspring hatched from the eggs laid, we collected 10 first instar nymphs from each pot (*N* = 160). We then prepared eight fresh pots with a filter-paper and randomly assigned each group of 10 R+ and 10 R– nymphs to a pot and presented them with the opportunity to feed every 3 days. As soon as the first 5th instar nymph was observed in a pot, eclosion was checked every day. Freshly eclosed adults were then removed from the pot and *post-hoc* screened for *Rickettsia* infection status using the PCR assays described above. The number of days between placement into the pot and the last hatching event, i.e., removal from the pot, represents the development time. The sex of individuals was determined when the bugs reached the adult stage. Sex ratio (number of female:male) was calculated and compared between the two infection status, R+ and R– individuals, which were identified with PCR assays as described above.

### Effect of *Rickettsia* Infection on Fecundity and Cytoplasmic Incompatibility

To measure the effect of *Rickettsia* infection on fecundity we used a full factorial crossing scheme of female x male, i.e., R+ × R+, R+ × R–, R– × R+ and R– × R–. For this, we prepared same-aged individuals by putting a 7-day-old virgin male and female together in a pot and allowing them to mate and feed weekly. Every week we collected all the eggs and put them in a fresh pot, which was fed weekly until 5th instar nymphs were observed. Same-aged 5th instars were then fed and placed into a 96-well plate until they reached adulthood. Seven-day-old virgin adults were then used in a full factorial crossing experiment. Prior to the experiment, females were fed twice, the last time on the day of mating, and males once, on the day of hatching. To avoid inbreeding effects, each isofemale line was crossed with every other line, but not with itself. To have equal sample sizes for within vs. between R+ and R– crosses we randomly left one cross out. In this manner, we crossed each isofemale line with three R+ lines and with three R– lines (*N* = 96 crosses). Matings were staged, monitored and interrupted after 60 s as described earlier in Reinhardt et al. (2009a). Interrupted matings were conducted to standardize sperm number because of the linear relationship between copulation duration and sperm number (Siva-Jothy and Stutt, 2003). A standardized sperm number was desirable since spermatozoa trigger the release of an oviposition-stimulating hormone from the *corpora allata* (Davis, 1956) and could potentially influence lifespan through differential egg production. The use of 60 s standard copulation also allows comparability with other studies.

After mating, the females were kept individually in 15 ml plastic tubes equipped with a piece of filter paper for egg laying. Females were fed weekly and the number of fertile and infertile eggs counted in weekly intervals. Fertile and infertile eggs were distinguished following Reinhardt et al. (2009b). Fertile eggs are taut and whitish with visible eye spots of the developing embryo. Infertile eggs normally collapse soon after being laid and are grayish. The number of fertile eggs was used to investigate the fecundity of crosses.

To determine the occurrence of CI, we observed the number of fertile eggs produced by females from different crossing combinations (Sakamoto and Rasgon, 2006). When fertile eggs are laid, it implies that the embryos in the eggs have already passed the critical point of CI. It has been observed that about one-third of embryogenesis happens within the ovaries before the eggs are laid (Usinger, 1966). We expected that if *Rickettsia* induces CI in the bed bug, the proportion of fertile eggs will be lowest in the group that only males from *Rickettsia*-infected line were crossed (R– × R+).

### Statistical Analyses

The data were analyzed using the statistical platform R (version 3.6.1, 2019) (R Development Core Team, 2019) using the packages “lme4” (Bates et al., 2015). The analysis of development time and fecundity were done by fitting linear mixed-effects models (LMMs) using the “lmer” function, while sex ratio and cytoplasmic incompatibility were fitted in generalized linear mixed effect model (GLMMs) using “glmer” function with binomial family.

For the development time and sex ratio, we fitted “pot” as a random effect in mixed effect models, while “infection status” and bed bug “populations” were fitted as the fixed effect in all cases. The factor “sex” was also included as a fixed effect explaining development time. The ratio of “female: male” and “number of fertile: infertile eggs” were set as the response variable in the sex ratio and fecundity test, respectively. In fecundity and CI analyses, “infection status” was broken down into “male infection status” and “female infection status” as these two factors represented cross types (female x male; R+ × R+, R+ × R–, R– × R+, R– × R–), alongside the fixed effect “population.” Family of origin was modeled as a random effect.

Non-significant effects were removed from the models until we found the minimum adequate model. We performed Likelihood ratio test (LRT) by comparing the minimum adequate models of both LMMs and GLMMs with a null model using “anova” function, considering by χ^2^ with critical *p*-value at 0.05. The normality and homoscedasticity of residuals of the LMMs models were validated before the final interpretation.

## Results

### Torix *Rickettsia* Across Bed Bug Populations and Other Cimicids

DNA extractions from each *C. lectularius* population and all cimicid allies passed QC, with good amplicons in the COI amplification. Thirteen out of 21 *C. lectularius* populations tested positive to *Rickettsia* infection with both *16S rRNA* and *gltA* primers. These populations have their origin in Africa and Europe (Table 1). Both male and female individuals were found to be infected in most cases where infection was detected. In the initial screen, the female individual alone was scored as infected in populations S1 and BG1; however, infection in males was observed in both populations on deeper screening (Table 1). For the cimicid allies, PCR results indicated that only one species, *Afrocimex constrictus* in one subfamily Afrocimicinae, were positive for *Rickettsia* infection with all three samples scoring positive (Table 2). However, the rickettsial PCR only produced *gltA* amplicons for this species, despite repeated attempts at 16S amplification.

### Phylogenies

**Figure 1:**
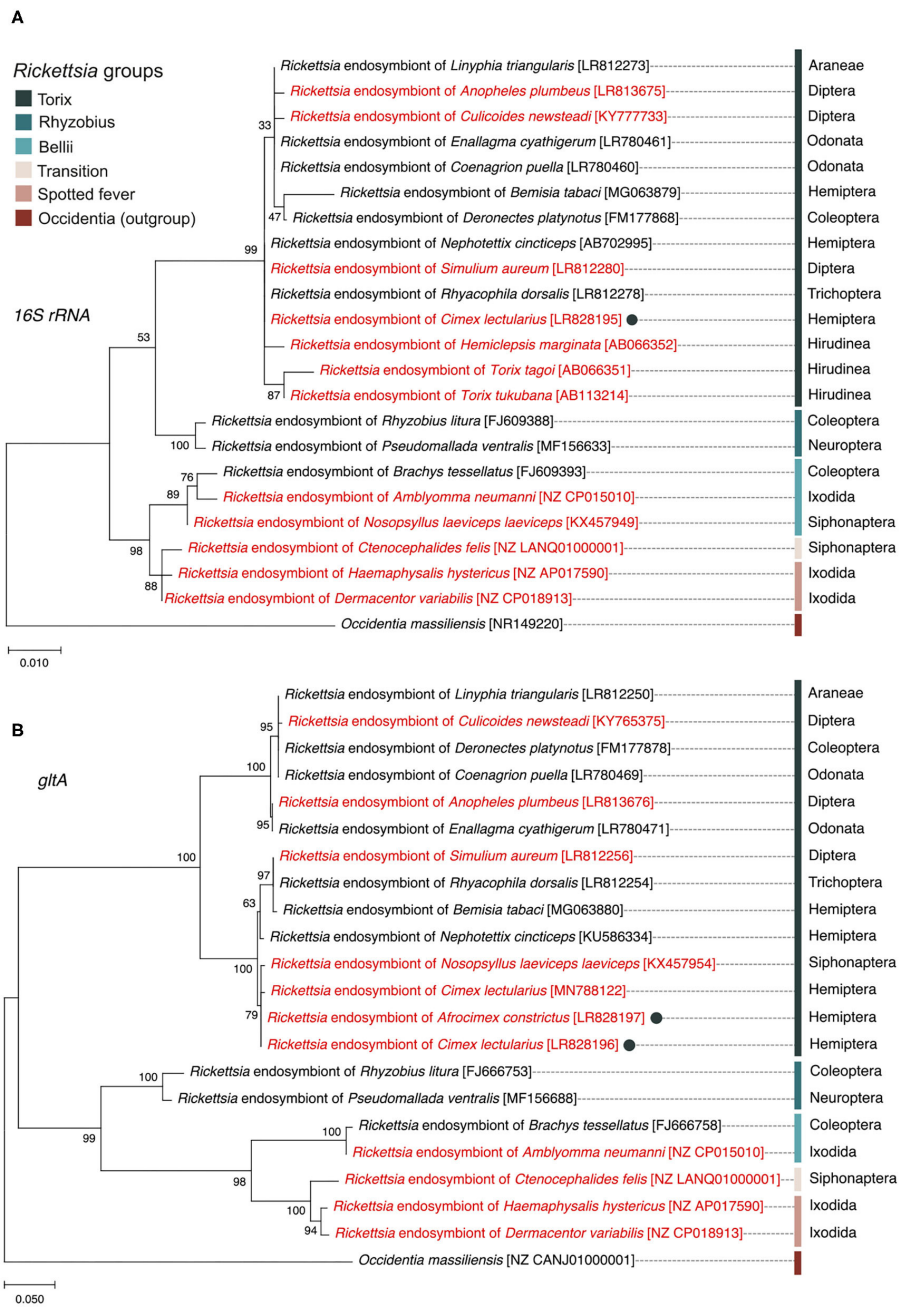
Maximum likelihood phylogenetic trees generated from *16S rRNA* gene **(A)** and *gltA* gene **(B)** sequences showing the position and relatedness of *Rickettsia* endosymbiont of *C. lectularius* and *A. constictus* found in this study (filled circles) in relation to other strains obtained from NCBI (the GenBank accession numbers are in the square brackets). *Rickettsia* from blood-feeder hosts are shown in red color. Scientific order names of arthropod hosts are provided at the end of the leaves while scientific class names are given for non-arthropod hosts. Bootstrap support values, expressed as the percentage of 1,000 replicates, are shown at the nodes. Bars indicate substitution nucleotide per position.

The *16S rRNA* sequence alignment of all *Rickettsia* strains from the *C. lectularius* populations indicated that these strains were identical (based on 985 bp sequence length information, accession number; LR828195). The phylogenetic tree based on *16S rRNA* gene showed the *Rickettsia* strain of *C. lectularius* is placed in torix group (Figure 1). The alignment of *gltA* sequences (746 bp) of *C. lectularius* also showed no variable sites across all *Rickettsia* positive populations. Moreover, the sequences of the *gltA* amplicons from both *C. lectularius* and *A. constrictus* (accession numbers; LR828196-LR828197) were also identical (Figure 1).

### Transmission Mode Experiment

**Figure 2:**
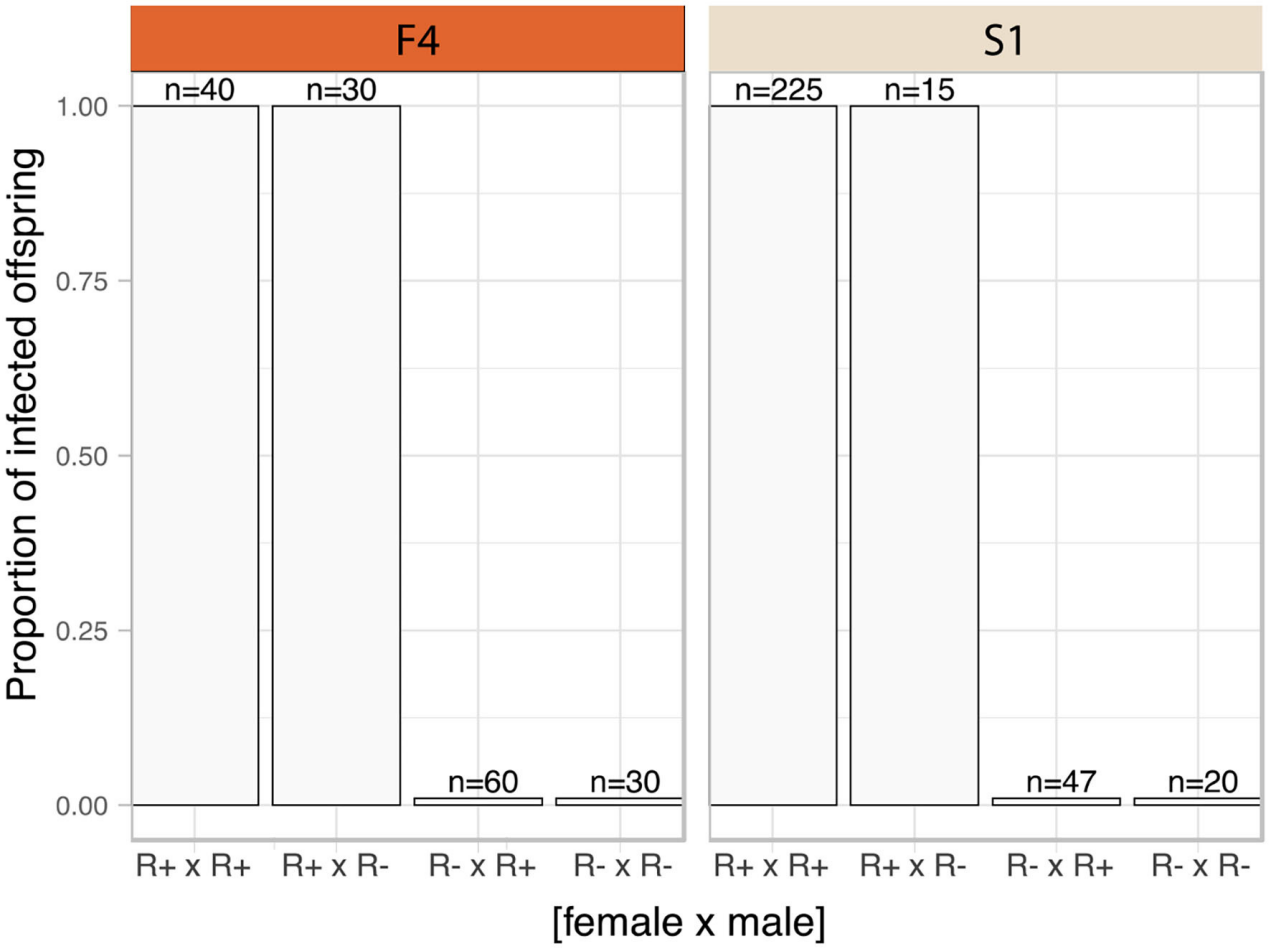
Transmission mode of *C. lectularius* associated *Rickettsia*. The bars indicate proportion of infected offspring in each crossing group. The four crosses were sorted by infection status of the parents, female × male (R+ × R+: 8 crosses for F4, 45 for S1; R+ × R–: 6 crosses for F4, 3 for S1; R– × R+: 12 crosses for F4, 5 for S1; for R– × R–: 23 crosses for F4, 2 for S1). No infected offspring were observed from *Rickettsia*-free mother groups (R– × R+ and R– × R–).

The frequency of infected parents in the F4 population was lower than that in S1 (F4: 14 of 49 mothers, and 20 of 49 fathers carried the symbiont; S1 population, 48 of 55 mothers, and 50 of 55 fathers carried the symbiont). The *Rickettsia* infection status for each family was categorized into four groups according to parental infection status. *Rickettsia* transmission to progeny was consistently observed where either both parents (R+ × R+) or just the mother (R+ × R-) tested positive for torix *Rickettsia* (R+ × R+: F4: 8 crosses, S1: 45 crosses; R+ × R-: F4: 6 crosses, S1: 3 crosses) (Figure 2). In these cases, all 310 tested nymphs tested positive for infection, indicating vertical transmission through females was highly efficient (Confidence intervals for vertical transmission efficiency 0.988–1.000). No progeny tested positive for *Rickettsia* infection in families where only the father was infected (R– × R+: F4: 12 crosses, S1: 5 crosses), nor were any *Rickettsia* positive individuals recovered from crosses where neither parent was infected (R– × R–: F4: 23 crosses, S1: 2 crosses). These data indicate that maternal infection is necessary and sufficient for presence of *Rickettsia* in progeny, and there is no evidence of paternal inheritance. The PCR assay for the BEV-like symbiont revealed that all individuals within these crosses were infected with this symbiont.

### Localization of the Symbionts

**Figure 3:**
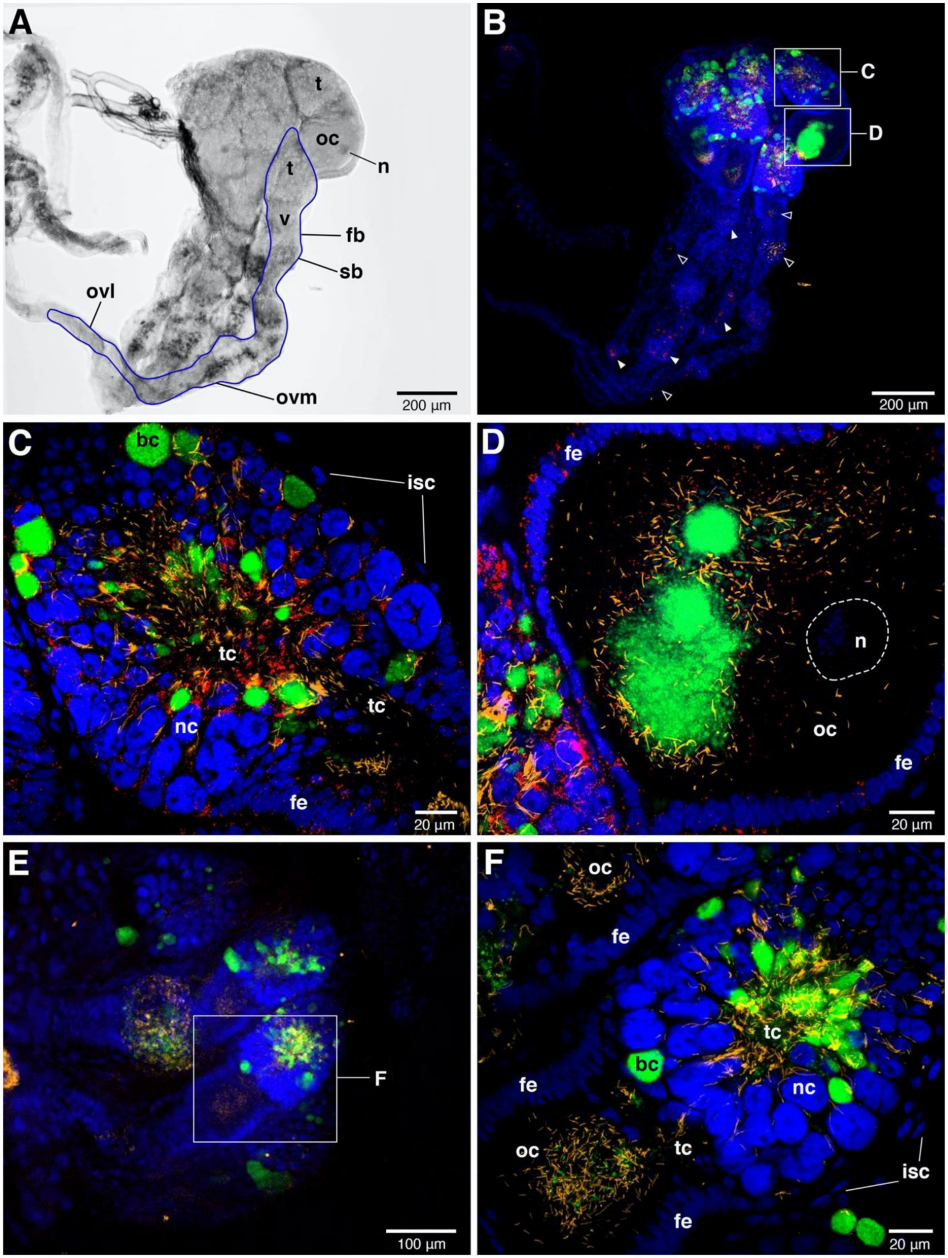
FISH images of adult female ovaries. **(A)** Bright field image of one-sided ovaries from *Rickettsia*-infected female. The blue line represents the outline of one ovariole. n = nucleus of oocyte, t = tropharium part, v = vitellarium part, fb = follicular body, oc = oocyte, ovl = lateral oviduct, ovm = mesodermal oviduct, sb = syncytial body. **(B)** FISH shows the presence of the three symbionts, i.e., *Wolbachia* (green), BEV-like symbionts (yellow), and *Rickettsi*a (red) in ovaries. Blue color represents nuclei of bed bug cells. The signals of three symbionts are concentrated in tropharium areas. Small rectangles indicate the magnified fields of tropharium and vitellarium portions, showing in **(C,D)**, respectively. *Rickettsia* (filled triangles) and BEV-like symbionts (open triangles) are also detected in syncytial body and mesodermal oviduct at low densities. **(C)** Enlarged detail of the tropharium portion. It is covered by a membrane of inner sheath cells (isc). The three symbionts distribution can be detected at very high density in all along the trophic core (tc) area. *Wolbachia* are likely packed in bacteriocytes (bc) which are distinctive to the adjacent nurse cells (nc), while *Rickettsia* and filamentous BEV-like symbionts are more scattered. **(D)** Enlarged detail of vitellarium portion. All three symbionts invade the oocyte, forming a cluster at the posterior pole of the oocyte. *Rickettsia* signals are scattered insertions in the follicular epithelium (fe) of the oocyte. **(E)** Ovaries of *Rickettsia*-free bed bug. Only *Wolbachia* and BEV-like symbionts are present. The small rectangle represents the tropharium and vitellarium parts of one ovariole, showing in **(F)**. **(F)** Enlarged field of partial upper ovariole. The distribution of *Wolbachia* and BEV-like symbionts are intensive in trophic core and in oocyte. None *Rickettsia* signals are detected here.

**Figure 4:**
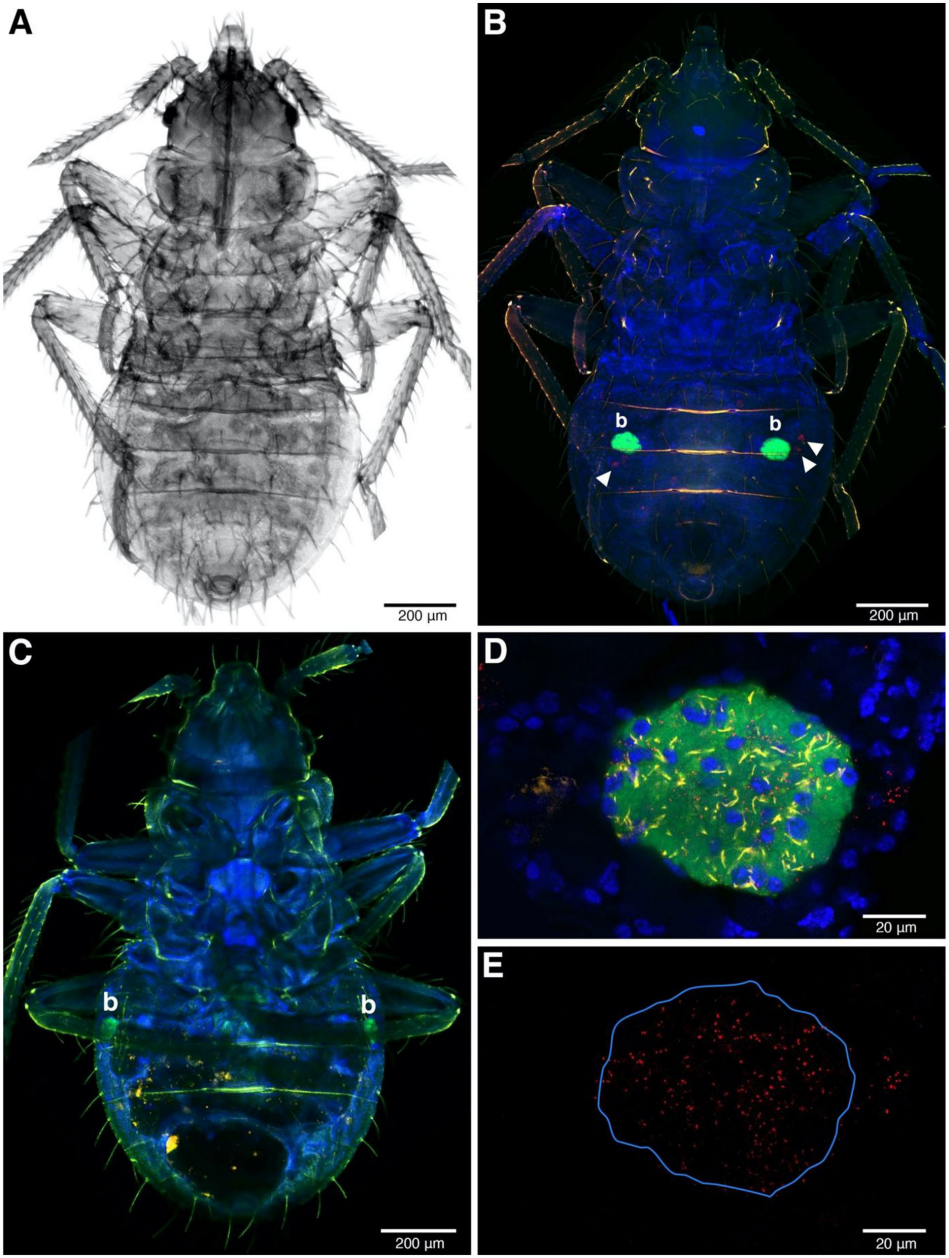
FISH image of whole-mounted first instars. **(A)** The nymph under transmitted light. **(B,C)** FISH detection of symbionts in *Rickettsia*-infected **(B)** and *Rickettsia*-free instars **(C)**. The ball-shaped with green fluorescent color represents strong *Wolbachia* signals, indicating the bacteriome (b) position in the abdomen. *Rickettsia* infection in red (filled triangles) present in **(B)** but absent in **(C,D)** Bacteriome of *Rickettsia*-infected instar. All the three symbionts can be detected in this organ. *Wolbachia*, BEV-like symbionts and *Rickettsia* are in green, yellow, and red, respectively. The blue color represents nuclei of bed bug cells. **(E)** The same field as **(D)** but only the *Rickettsia* channel remains. The blue line indicates the bacteriome boundary.

FISH detected *Rickettsia* throughout ovaries and bacteriome tissues (Figures 3, 4). In adult females, the distribution of the *Rickettsia* signal was intense in the core of the tropharium and in oocytes. We observed the filamentous BEV-like symbiont and *Wolbachia* alongside *Rickettsia* in this tissue. In comparison to the other symbionts, *Rickettsia* were more widely distributed in the ovaries. We observed them additionally in nurse cells and follicular epithelial cells (Figures 3C,D). *Rickettsia* signals were absent in R- bed bugs (Figures 3E,F). The pattern of *Rickettsia* infection was similar between the F4 and S1 populations.

*Rickettsia* signals were found in somatic tissue of the abdominal areas of first instar whole mounts, alongside the BEV-like symbiont (Figure 4B). At low magnification, the strongest signal (green color) was emitted by *Wolbachia*, reflecting the intense infection of bacteriome organs (Figure 4B). *Rickettsia* and the BEV-like symbiont, however, could also be seen at this low magnification but poorly resolved. At the higher magnification, *Rickettsia* is clearly visible in the bacteriome alongside *Wolbachia* and the BEV-like symbiont as all the three signals were reliably present in this tissue (Figures 4D,E). Again, a similar pattern of *Rickettsia* infection was observed in F4 and S1 populations. In R– samples, only the signals of *Wolbachia* and the BEV-like symbiont were present, while *Rickettsia* signals were absent (Figure 4C).

### Development Time and Sex Ratio

**Figure 5:**
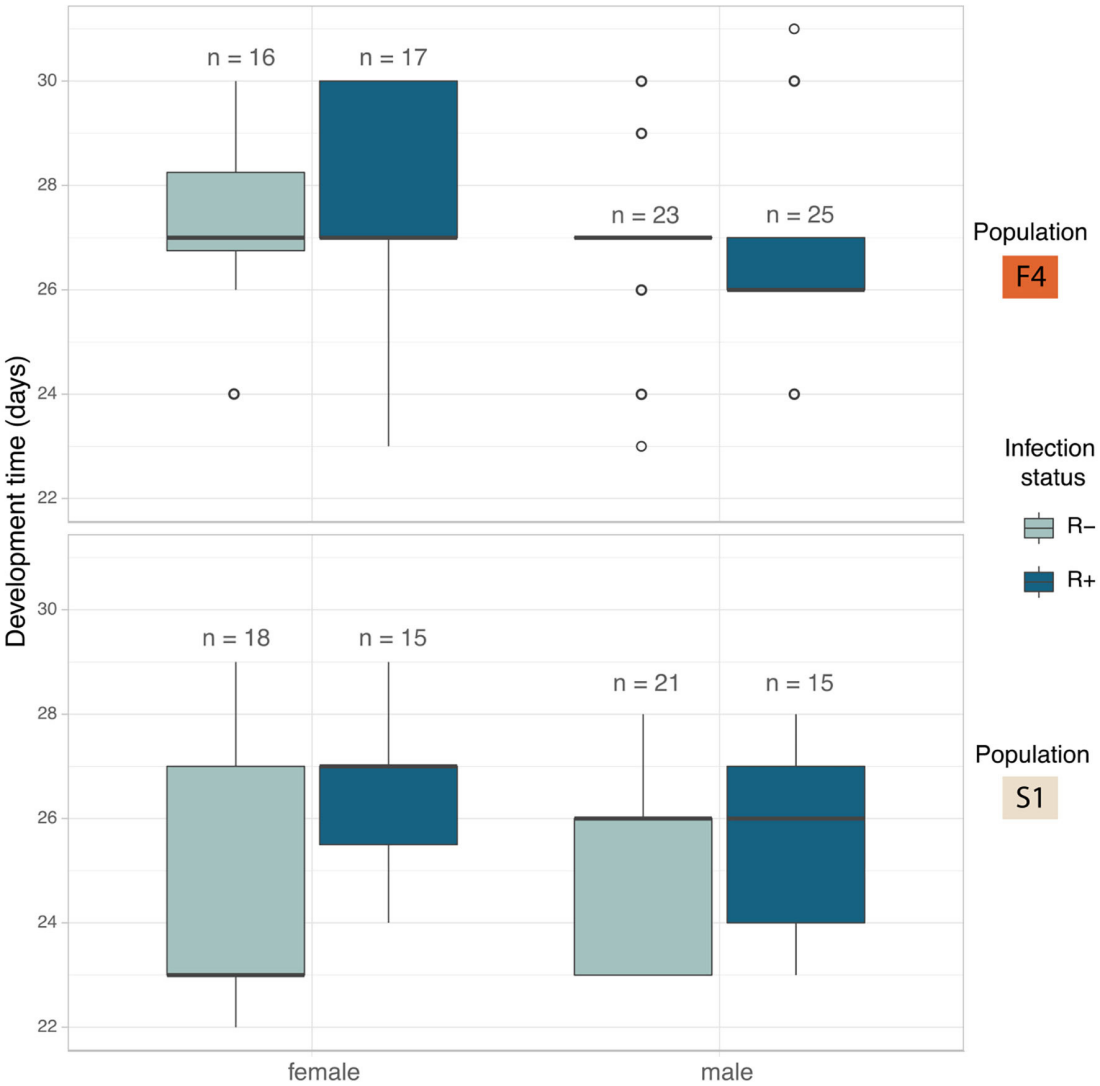
Median development time in days of *C. lectularius* from the first instar to adulthood for males and female individuals of *Rickettsia*-free (R–, light blue) and *Rickettsia*-infected (R+, dark blue) groups from population F4 (top) and S1 (below). *Rickettsia* infection has a significant effect on development time (LRT: χ^2^(1) = 5.177, *p* = 0.023). Boxes indicate the 25 and 75 percent quartiles, respectively, the whiskers show minimum and maximum values. Open circles indicate potential outliers.

Development showed a different pattern between the populations. In F4 the development times were similar across all four crosses (Figure 5). S1 showed a substantial difference between the development time of R+ and R– females, whereas R+ and R– males were the same (Figure 5). The full model analysis (including interaction terms between sex, population and *Rickettsia* infection status) was not a significantly better fit than the model without interaction terms (LRT χ^2^(4) = 5.554, *p* = 0.235).

The impact of individual terms was then examined in models without an interaction term. The only significant explanatory variable was “infection”: LRT of models with and without infection term: χ^2^(1) = 5.177, *p* = 0.023). First instar-adult period for *Rickettsia* infected individuals increased by 0.59 ± 0.26 days (Mean ± SD). The *Rickettsia*-infected line took 26.7 ± 2.00 days to reach adulthood (*N* = 78) while the bugs from the *Rickettsia*-free line took 25.9 ± 2.15 days (*N* = 72) (Figure 5). Population and sex were not observed to explain variation in development time.

**Figure 6:**
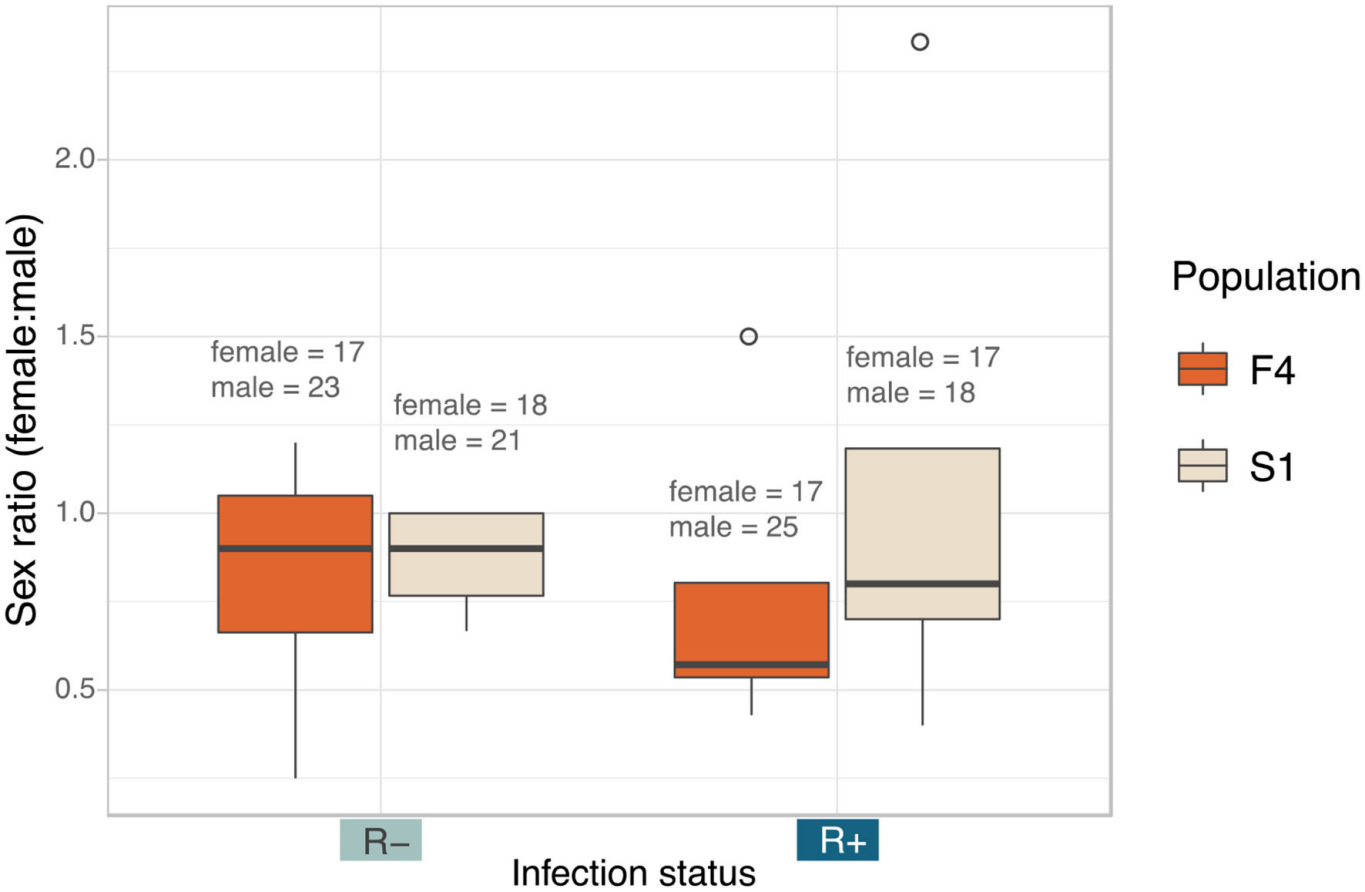
Median sex ratio (number of female:male) of the *Rickettsia*-free and *Rickettsia*-infected *C. lectularius* adults from the two populations. The sexes were identified from adult bed bugs. There was no significant different of the sex ratio between R– and R+ group at *p* = 0.05. Total number of males and females are shown above the boxes. Boxes indicate the 25 and 75 percent quartiles, respectively, the whiskers show minimum and maximum values. Open circles indicate potential outliers.

There was no impact of *Rickettsia* infection status on the sex ratio of offspring (LRT: χ^2^(1) = 0.0003, *p* = 0.985) (Figure 6). Female: male ratio of the *Rickettsia*-free group was 0.84 ± 0.29 and 0.93 ± 0.67 for the *Rickettsia*-infected group. The interaction effect between infection status and population of origin was not statistically significant (LRT: χ^2^(1) = 0.078, *p* = 0.780).

### Fecundity and Cytoplasmic Incompatibility

**Figure 7:**
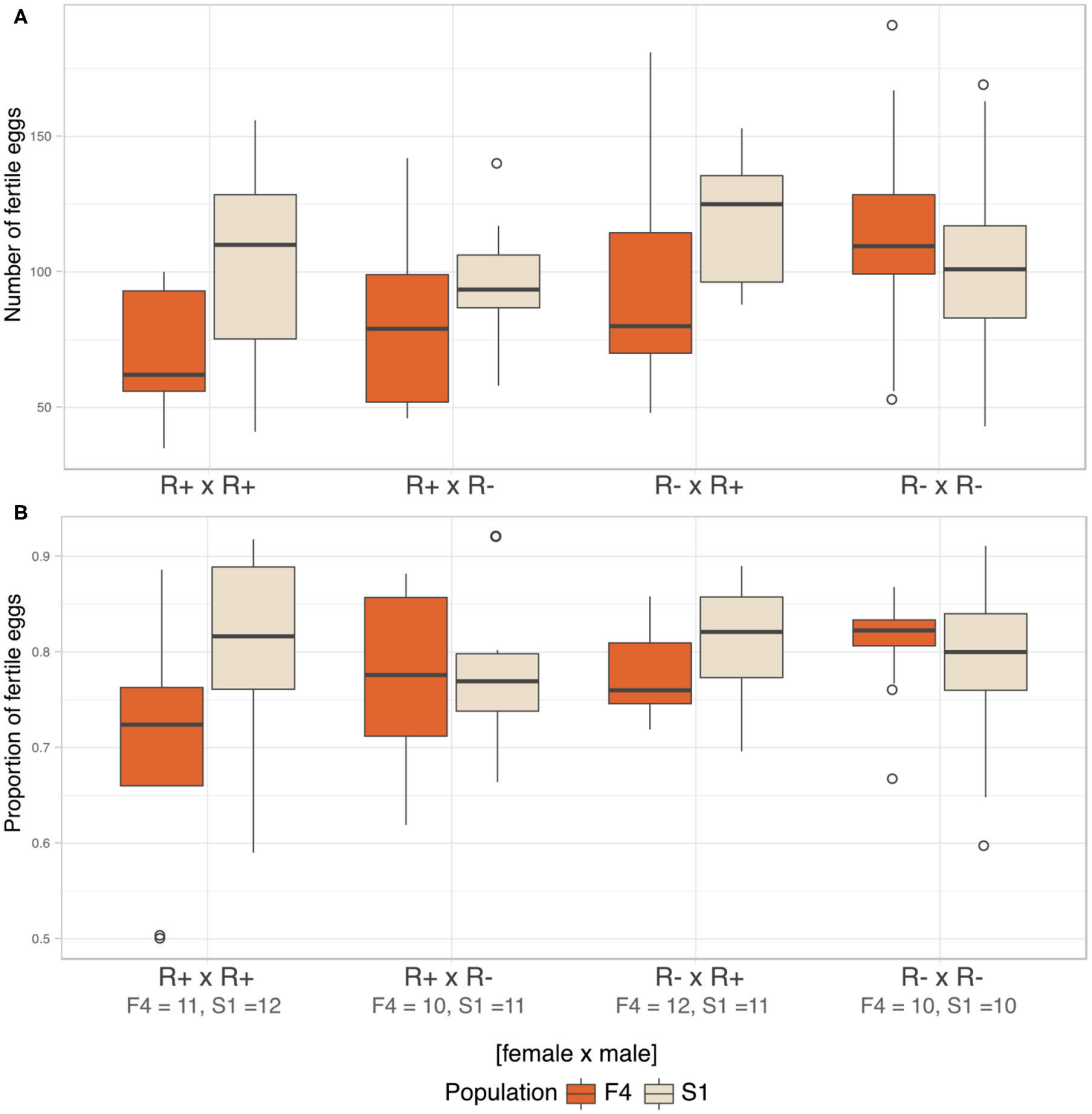
Fecundity and CI. **(A)** Median number of fertile eggs of *C. lectularius* from population F4 and S1 from the four cross combinations. *Rickettsia*-infected female crosses (R+ × R+ and R+ × R–) produced significantly fewer fertile eggs when compare to other crosses (LRT: χ^2^(1) = 4.576, *p* = 0.032). **(B)** Median proportion of fertile eggs of *C. lectularius* from the two populations. There was no interaction effect of male and female infections on the ratio of fertile:infertile eggs from the GLMMs analysis at *p* = 0.05, indicating there was no evidence of CI. Number of crosses completed are shown under the cross group. Boxes indicate the 25 and 75 percent quartiles, respectively, the whiskers show minimum and maximum values. Open circles indicate potential outliers.

We analyzed fecundity (total fertile eggs) using likelihood ratio comparison of LMMs. There was no evidence of an interaction effect between the three factors, i.e., population, male, and female infection status (LRT: χ^2^(3) = 5.781, *p* = 0.216), and these terms were dropped from the model. The final model detected *Rickettsia* infection in female parent as the sole significant explanatory variable for fecundity (LRT: χ^2^(1) = 4.576, *p* = 0.032). Infected females were likely to produce fewer fertile eggs (R+ × R+ = 86.80 ± 34.30, R+ × R– = 89.70 ± 28.30) compared to non-infected females (R– × R+ = 107.00 ± 34.90, R– × R– = 109.00 ± 34.60, Figure 7A).

We then analyzed the relative ratio of fertile:infertile eggs to ascertain if there was any evidence of cytoplasmic incompatibility. There was no evidence of heterogeneity associated with *Rickettsia* infection in either male (LRT: χ^2^(1) = 0.593, *p* = 0.441) or female parents (LRT: χ^2^(1) = 1.174, *p* = 0.279). There was no evidence of an interaction term between male infection x female, evidence by the statistical equivalence of models with and without an interaction term (LRT: χ^2^(4) = 6.725, *p* = 0.151, Figure 7B). However, the number and proportion of fertile eggs was higher in F4 when the males were R– whilst the opposite was the case for S1, where both variables were higher when the males were R+.

## Discussion

Torix *Rickettsia* are common associates of insects and other invertebrates, but their mode of transmission and impact on the host are poorly understood. In the present study, we examined the interaction between torix *Rickettsia* and *C. lectularius*, a notorious pest of humans. In line with other recent work, our PCR screen revealed *Rickettsia* were commonly found in bed bug lines maintained in the laboratory with only a single *Rickettsia* strain detected. Segregation of the *Rickettsia* in two lines was observed – with a mix of infected and uninfected individuals being observed in laboratory populations. The polymorphism in *Rickettsia* presence allowed us to isolate isogenic R+ and R– cultures and then use these to analyse transmission and impact on the host. We observed that the symbiont showed high fidelity maternal inheritance, but no transmission through males. Consistent with this, *Rickettsia* infections were present in ovaries, as well as in the bacteriomes. Infection with this symbiont did not impact host sex ratio and did not induce cytoplasmic incompatibility, but there was evidence it slowed development to the adult stage and reduced female fecundity.

In this study, the *Rickettsia* strain detected in *C. lectularius* and *A. constrictus* are placed in the non-vertebrate pathogen “torix group” which consisted of *Rickettsia* endosymbionts of other arthropods, e.g., *Deronectes* diving beetle, *Rhyacophila* caddisfly, *Linyphia* spider, *Nosopsyllus* flea, *Anopheles* mosquito, *Culicoides* biting midge, and other non-arthropod hosts, e.g., glossiphoniid leeches (*Hemiclepsis marginata, Torix tukubana*, and *T. tagoi*) (Figure 1). Hosts of torix clade *Rickettsia* are biased toward species that are either aquatic (diving beetle and caddisfly), blood feeding (bed bug and flea), or both aquatic and blood feeding (mosquito and leeches). However, the pair of loci we sequenced are not sufficient to resolve whether there is any pattern to symbiont distribution amongst host species, and analysis of this awaits a wider MLST study. The detection of a single torix *Rickettsia* strain in cosmopolitan bed bug species indicates that one main strain of this symbiont circulates in this species worldwide, consistent with movement alongside travelers. This finding also appears in the recent study of Potts et al. (2020). These *Rickettsia* strains were investigated in *C. lectularius* from the UK, which are independent from the populations in our study, and field collections from the USA, all of which reveal an identical *gltA* haplotype.

It is typical for inherited endosymbionts to be transmitted maternally between host generations (Hosokawa et al., 2010; Touret et al., 2014; Pilgrim et al., 2017; Feng et al., 2019; Rosenwald et al., 2020; Thongprem et al., 2020b). Similarly, our bed bug associated torix *Rickettsia* was observed to be maternally inherited. In the present experiments, maternal inheritance reliably occurred with all 310 tested offspring carrying the *Rickettsia*. However, evidence of segregation was observed in population F4 over the last 10–12 years of laboratory passage. *Rickettsia* infection in the F4 bed bug DNA materials from Duron et al. (2008) was complete, but was found in just 35% of individuals in 2019 (ca. 100 bug generations later). Thus, whilst transmission fidelity is high, there is a low level of loss during maternal inheritance. Thus, we can conclude that whilst vertical transmission through females is very high, it does not occur with a 100% efficiency.

In contrast, no paternal transmission was observed in this study, despite paternal males carrying infections, and previous evidence of *Rickettsia* in male sperm vesicles (Bellinvia et al., 2020). The situation contrasts with the leafhopper-associated *Rickettsia*. In *Nephotettix cincticeps*, torix *Rickettsia* can transmit biparentally, with 70% paternal and 100% maternal transmission rates. The *Rickettsia* are found in sperm without interrupting sperm function (Watanabe et al., 2014). Paternal inheritance has also been noted for symbionts in the genus *Megaira*, the sister taxa to *Rickettsia* (Kochert and Olson, 1970). Notably, the *Rickettsia* in the leafhopper has the capacity for intranuclear infection, which likely is necessary for paternal inheritance. Beside this, paternal transmission is also observed in a tsetse fly-associated *Sodalis* symbiont (De Vooght et al., 2015). Intranuclear infection is present in several endosymbiotic bacteria, e.g., *Nucleococcus, Chlamydia*, gamma and alpha proteobacteria (Schulz and Horn, 2015). This trait is observed quite widely in the Rickettsiacae, but is labile, being present in some symbioses but not others (Schulz and Horn, 2015).

Insects commonly live mutualistically with endosymbionts. In many cases, insect hosts provide a pair of bacteriomes as organs for harboring their endosymbionts (Douglas, 1989; Baumann et al., 2006; Hosokawa et al., 2010; Noda et al., 2012; Marubayashi et al., 2014). Previous histological studies have located the bacteriome organs at either side of the bed bug abdomen. So far, only two bacteriome-associated endosymbionts have been described in *C. lectularius*, i.e., the primary endosymbiont *Wolbachia* and a secondary BEV-like symbiont (Hosokawa et al., 2010). Here we additionally examined the tropism of *Rickettsia*, alongside *Wolbachia* and the BEV-like symbiont. Overall, the bacteriome FISH image indicates a high signal intensity for *Wolbachia*. *Rickettsia* was also present in the bacteriome and the three symbionts were spatially intermixed (Figure 4D). This result contrasts with the localization of the symbiont community in the leafhopper *N. cincticeps*, in which the different symbiont species live separately. The facultative *Rickettsia* is widely dispersed through *N. cincticeps* bacteriomes and can be found in most of the leafhopper tissues (Mitsuhashi and Kono, 1975). When the symbiont infection has a specific territory within the bacteriome this implies a more specific function of those symbionts for their host (Nakabachi et al., 2013; Dan et al., 2017). Investigating inside the bed bug bacteriomes might help to understand the distribution of these three endosymbionts and could potentially anticipate the biological impacts of these endosymbionts on the host or their interaction.

The presence of *Rickettsia* in bacteriocytes indicates a route for achieving maternal inheritance. In this symbiosis, vertical transmission of *Wolbachia* is associated with the movement of bacteriocytes toward the ovary, where they fuse with oocytes to deliver the symbiont. The presence of *Rickettsia* in the bacteriome likely allows this symbiont to hitch-hike to the ovary to gain vertical transmission. However, having established in the ovary, *Rickettsia* and *Wolbachia* show distinct patterning, with *Wolbachia* clustering in discrete clumps and *Rickettsia* being more dispersed. Although this study lacks a visual evidence of *Rickettsia* infection in other somatic tissues (e.g., haemolymph, excretory, digestive, and immune systems), the consistent detection of *Rickettsia* infection in head and leg materials during the transmission experiment indicate the infection is diffusely present. *Rickettsia* infection in legs is likely to be derived from haemolymph, as has been reported in other *Rickettsia*-host systems (Hurst et al., 1996; Caspi-Fluger et al., 2011; Shan et al., 2020).

We also examined the impact of *Rickettsia* on bed bug biology. We found no evidence of reproductive parasitic phenotypes (sex ratio distortion or cytoplasmic incompatibility). There was evidence of a weak negative impact on first instar-adult development time, and also reduced fecundity of *Rickettsia* infected females. *Rickettsia* impact differed between the two populations tested. This may be a product of differences in symbiont or host genomes, or an interaction between the two. Overall, all metrics that we examined showed either no evidence of an effect of *Rickettsia* on the trait, or a deleterious impact. The results are similar to those observed in the *Spalangia*-*Rickettsia* interaction, where *Rickettsia* (again in the presence of *Wolbachia*) was associated with a 1 day developmental delay, but did not induce changes in either sex ratio or CI (Semiatizki et al., 2020).

The combination of segregational loss and modest costs indicate maintenance of the symbiont in field populations will require some balancing benefit. A recent example of the biological impact of torix *Rickettsia* can be found in glossiphoniid leeches-associated *Rickettsia*. The case study demonstrates that *Rickettsia* have a direct effect on the body size of the three leech host species, with infected individuals being larger (Kikuchi and Fukatsu, 2005). A recent study has examined the genome sequence of torix *Rickettsia* endosymbiont of *Culicoides newsteadi*, a strain closely related to bed bug-associated *Rickettsia* (Pilgrim et al., 2017). No evidence of the capacity for positive facilitation was observed in the genome, e.g., B-vitamin provisioning. However, the genome possesses unique features of genes that are potentially associated with host invasion and adaptation (Pilgrim et al., 2017). It is likely that any benefits of *Rickettsia* infection are ecologically contingent, and parallel with other symbioses indicating resistance to environmental stress or pathogen susceptibility as worthwhile avenues for research.

## Data Availability Statement

The datasets presented in this study can be found in online repositories. The names of the repository/repositories and accession number(s) can be found in the article/Supplementary Material.

## Author Contributions

The study was initially devised by PT and GH and experiments designed by PT, SE, GH, and OO. Rearing of bed bugs was completed by SE and OO. Screening of bed bugs, marker sequencing, and FISH analysis was completed by PT with advice from GH. Analysis of developmental time, sex ratio, fecundity, and incompatibility were completed by OO. PT, OO, and GH wrote the manuscript and all authors contributed to the drafting.

## Conflict of Interest

The authors declare that the research was conducted in the absence of any commercial or financial relationships that could be construed as a potential conflict of interest.
