# Supplementary material for: Transmission, Tropism, and Biological Impacts of Torix Rickettsia in the Common Bed Bug Cimex lectularius (Hemiptera: Cimicidae)
Source: Front Microbiol. 2020 Dec 23;11:608763. doi: 10.3389/fmicb.2020.608763 (PMC7785988; doi:10.3389/fmicb.2020.608763)
Supplement: Supplementary file 1 [file Table_1.docx]

**Supplementary Material**

**Thongprem et al. Transmission, tropism and biological impacts of torix *Rickettsia* in the common bed bug *Cimex lectularius* (Hemiptera: Cimicidae)**

***Frontiers in Microbiology***

**Data deposition:**

Sequences have been deposited in EMBL (Accession numbers; LR828195- LR828197).

Data underpinning Figures 5-7 can be accessed at <https://doi.org/10.6084/m9.figshare.c.5127290.v1>

**Table S1** PCR primer and fluorescence probe sequences that were used in this study. All the fluorescence probes are labelled with fluorophore, in the square brackets, at 5’ end except *RickB1* probe, labelled at 3’ end. All the primers were used in the following PCR conditions; initial denaturation at 95 °C for 5 min, followed by 35 cycles of denaturation (94°C for 30s), annealing (Tm°C for 30s), extension (72°C for 50s), and a final extension at 72°C for 7 min. The annealing temperature was varied according to the primers.

| Target organisms:  gene | Primer/ probe Name | Sequence (5’-3’) | Tm (^o^C) | Product size (bp) | Ref. |
| --- | --- | --- | --- | --- | --- |
| Bed bug: *COI* | *C1J_1718* | GGA GGA TTT GGA AAT TGA TTA GT | 52 | 380 | (1) |
|  | *HCO_2198* | TAA ACT TCA GGG TGA CCA AAA AAT CA |  |  | (2) |
| *Rickettsia*: *16S rRNA* | *Ri170_F* | GGG CTT GCT CTA AAT TAG TTA GT | 54 | 1.1k | (3) |
|  | *Ri1500_R* | ACG TTA GCT CAC CAC CTT CAG G |  |  |  |
| *Rickettsia*: *gltA* | *RiGltA405_F* | GAT CAT CCT ATG GCA | 54 | 786 | (4) |
|  | *RiGltA1193_R* | TCT TTC CAT TGC CCC |  |  |  |
| BEV-like symbiont:  *16S rRNA* | *BEVF* | GCA CAA GGG AGG TTG CTC CCC | 57 | 420 | (5) |
|  | *BEVR* | CAG CAA GGT TAT TAA CCT TAC TG |  |  |  |
| BEV-like symbiont: rRNA probe | *CimexSec1229R* | [AlexaFluor555]-TTG CTC TCG CGA GGT CGC TT | - | - | (5) |
| *Rickettsia*: rRNA probe | *RickB1* | CCA TCA TCC CCT ACT ACA-[ATTO 633] | - | - | (6) |
| *Wolbachia*:  rRNA probe | *TsWo1187Rl* | [AlexaFluor488]-CTC GCG ACT TTG CAG CCC A | - | - | (5) |
|  | *TsWol944R* | [AlexaFluor488]-AAC CGA CCC TAT CCC TTC G | - | - |  |

References

1. Simon C, Frati F, Beckenbach A, Crespi B, Liu H, Flook P. Evolution, weighting, and phylogenetic utility of mitochondrial gene sequences and a compilation of conserved polymerase chain reaction primers. Annals of the Entomological Society of America. 1994;87(6):651-701.

2. Folmer O, Black M, Hoeh W, Lutz R, Vrijenhoek R. DNA primers for amplification of mitochondrial cytochrome c oxidase subunit I from diverse metazoan invertebrates. Molecular Marine Biology and Biotechnology. 1994;3(5):294-9.

3. Küchler SM, Kehl S, Dettner K. Characterization and localization of *Rickettsia* sp. in water beetles of genus *Deronectes* (Coleoptera: Dytiscidae). FEMS Microbiology Ecology. 2009;68(2):201-11.

4. Pilgrim J, Ander M, Garros C, Baylis M, Hurst GDD, Siozios S. Torix group *Rickettsia* are widespread in *Culicoides* biting midges (Diptera: Ceratopogonidae), reach high frequency and carry unique genomic features. Environ Microbiol. 2017;19(10):4238-55.

5. Hosokawa T, Koga R, Kikuchi Y, Meng X-Y, Fukatsu T. *Wolbachia* as a bacteriocyte-associated nutritional mutualist. PNAS. 2010;107(2):769-74.

6. Perotti MA, Clarke HK, Turner BD, Braig HR. *Rickettsia* as obligate and mycetomic bacteria. The FASEB Journal. 2006;20:E1646-56.
